# Supplementary material for: The interactome of intact mitochondria by cross-linking mass spectrometry provides evidence for coexisting respiratory supercomplexes
Source: Mol Cell Proteomics. 2017 Dec 8;17(2):216–32. doi: 10.1074/mcp.RA117.000470 (PMC5795388; doi:10.1074/mcp.RA117.000470)
Supplement: Supplemental Data [file supp_RA117.000470_133922_0_supp_23978_fzffwf.zip › spectra_annotation/mito_DR_spectra_annotation/463-1-12-1-2-1.pdf]

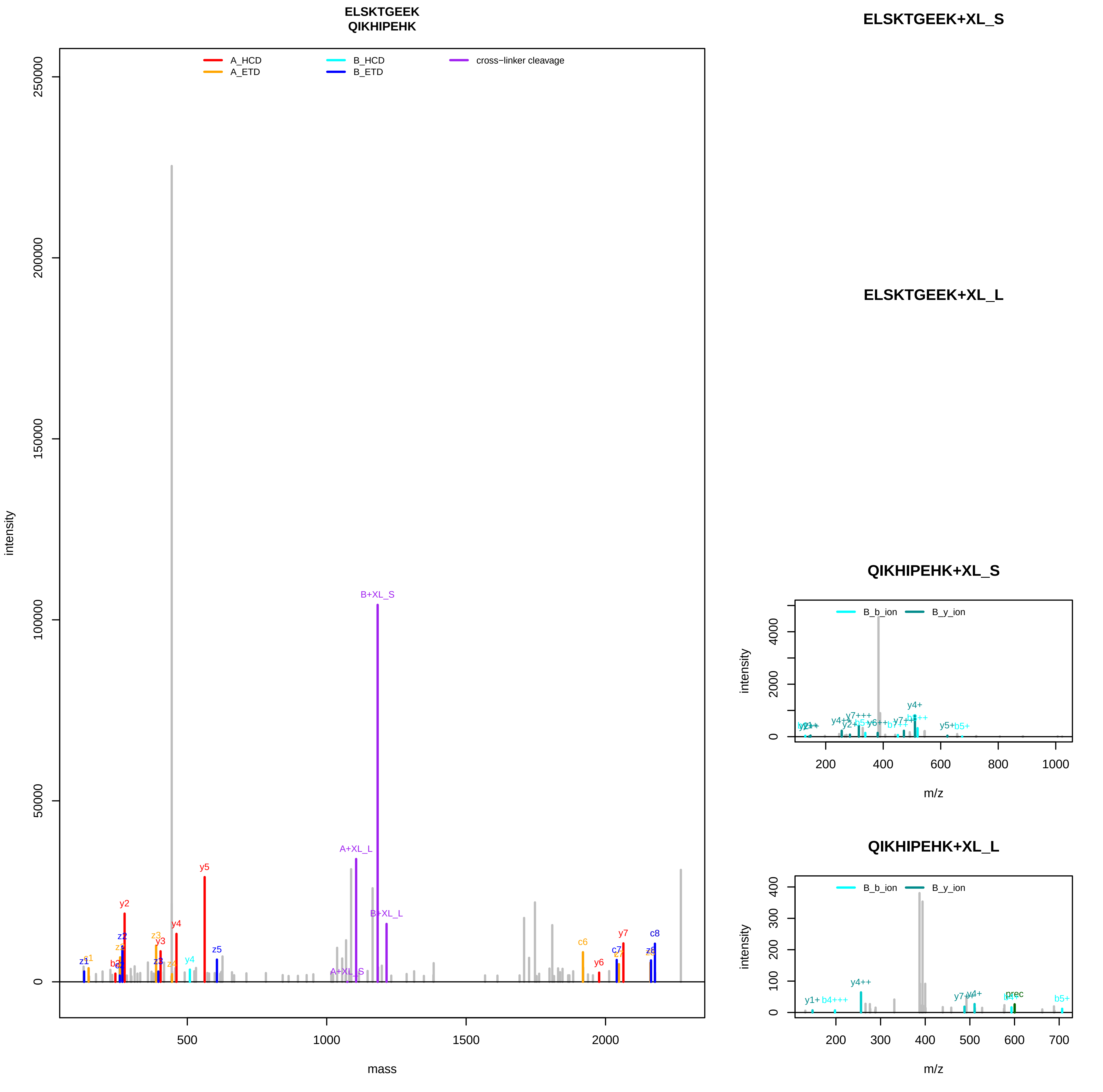

ELSKTGEEK
QIKHIPEHK
A_HCD
A_ETD
B_HCD
B_ETD
cross−linker cleavage
intensity
mass
0
50000
100000
150000
200000
250000
500
1000
1500
2000
B+XL_S
A+XL_L
B+XL_L
A+XL_S
y2
y3
y4
y5
y6
y7
z1
z2
z3
z4
z5
z7
z8
c6
c7
c8
ELSKTGEEK+XL_S
ELSKTGEEK+XL_L
QIKHIPEHK+XL_S
B_b_ion
B_y_ion
intensity
m/z
0
2000
4000
200
400
600
800
1000
y4+
y5+
b5+
QIKHIPEHK+XL_L
B_b_ion
B_y_ion
intensity
m/z
0
100
200
300
400
200
300
400
500
600
700
y1+
b4+++
y4++
y4+
prec
b5+
